# Intestinal symptoms and psychological factors jointly affect quality of life of patients with irritable bowel syndrome with diarrhea

**DOI:** 10.1186/s12955-015-0243-3

**Published:** 2015-04-18

**Authors:** Liming Zhu, Dan Huang, Lili Shi, Liexin Liang, Tao Xu, Min Chang, Wei Chen, Dong Wu, Facan Zhang, Xiucai Fang

**Affiliations:** Department of Gastroenterology, Peking Union Medical College Hospital, Chinese Academy of Medical Sciences and Peking Union Medical College, 1# Shuaifuyuan, Dongcheng District, Beijing, 100730 China; Department of Psychological Medicine, Peking Union Medical College Hospital, Chinese Academy of Medical Sciences and Peking Union Medical College, 1# Shuaifuyuan, Dongcheng District, Beijing, 100730 China; Department of Epidemiology and Statistics, Institute of Basic Medical Sciences, Chinese Academy of Medical Sciences & School of Basic Medicine, Peking Union Medical College, Beijing, China; Department of Gastroenterology, The People’s Hospital of Guangxi Zhuang Autonomous Region, Nanning, China

**Keywords:** Irritable bowel syndrome with diarrhea, Quality of life, Psychological factors, Intestinal symptoms, Gender

## Abstract

**Background:**

Patients with irritable bowel syndrome (IBS) have significantly reduced quality of life (QOL). Although intestinal and extraintestinal symptoms, as well as comorbid psychological disorders, may reduce the QOL of IBS patients, the primary determinant of QOL in these patients remains unclear. This study aimed to identify the main factors affecting QOL in patients with IBS with diarrhea (IBS-D).

**Methods:**

Consecutive patients meeting the Rome III Diagnostic Criteria for IBS-D were enrolled in this study. Patients with organic diseases were excluded. The intestinal symptoms, psychological states and QOL of these patients were evaluated using IBS-specific symptom questionnaires, the Hamilton Depression Scale (HAMD), the Hamilton Anxiety Scale (HAMA), and the Chinese version of the IBS-QOL instrument. Overall scores for intestinal symptoms were calculated by frequency and degree.

**Results:**

This study enrolled 227 IBS-D patients, of mean age 44.68 ± 10.81 years. Their mean overall IBS-QOL score was 71.68 ± 18.54, with the lowest score being for food avoidance (53.71 ± 26.92). Overall IBS-QOL score correlated negatively with overall scores of intestinal symptoms and HAMD and HAMA scores (p < 0.001 each). Overall intestinal symptoms scores correlated negatively with HAMD and HAMA scores (p < 0.001 each). Scores of HAMD, HAMA and structural factors (i.e., anxiety/somatization, cognitive disorder, psychomotor retardation, psychic anxiety, and somatic anxiety) were significantly higher in female than in male patients (p < 0.01). Food avoidance and social reaction scores of female patients were significantly lower than those of male patients (p < 0.05 each). The degree of defecation urgency, frequency of passing mucus and psychomotor retardation were independent factors predicting reduced QOL in IBS-D patients.

**Conclusion:**

Intestinal symptoms and psychological factors jointly reduce the QOL of IBS-D patients, with gender differences in the impact of both factors on QOL.

## Background

Irritable bowel syndrome (IBS) is characterized by abdominal pain or discomfort associated with alterations in bowel habits and stool forms. The symptoms are recurrent or persistent, which can significantly affect patients’ quality of life (QOL). QOL has been reported to be significantly lower in IBS patients than in patients with chronic diseases such as diabetes mellitus, hypertension, and chronic renal failure [1,2]. QOL in IBS patients may be affected by both intestinal and extraintestinal symptoms, as well as by comorbid psychological disorders. Although studies have investigated the QOL status of IBS patients, including analyzing the impact of intestinal symptoms and/or psychological states on patients’ QOL [3-6], the major determinant of QOL in these patients has not been determined.

The Rome III Diagnostic Criteria for Functional Gastrointestinal Disorders classified IBS into four subtypes: IBS with diarrhea (IBS-D), IBS with constipation (IBS-C), mixed IBS (IBS-M) and unsubtyped (IBS-U), with no significant differences in QOL among these four subtypes [4]. In China, IBS-D is the most common subtype, with prominent clinical symptoms and a high rate of hospital visits [7,8]. This study therefore investigated the associations of intestinal symptoms and psychological status with QOL, as well as determining the main factors affecting QOL in patients with IBS-D.

## Methods

### Subjects

This study included consecutive patients diagnosed with IBS based on the Rome III IBS Diagnostic and Classification Criteria between July 2009 and October 2011 and aged 18 to 70 years. Patients with organic gastrointestinal diseases, connective tissue diseases and metabolic diseases were excluded based on the results of routine blood, urine, stool, and fecal occult blood tests; liver, kidney, and thyroid function tests; measurements of carcinoembryonic antigen, erythrocyte sedimentation rate, and C-reactive protein; and abdominal ultrasound and colonoscopy, performed within the past year. All included patients participated in the study voluntarily and provided written informed consent. This study was approved by the Ethics Committee of Peking Union Medical Collage Hospital on July 7, 2009 (S-234).

### Questionnaires

#### IBS symptom questionnaire and scoring of overall intestinal symptoms

The questionnaire was developed based on our previous studies of IBS epidemiology [9] and modified based on Rome III: the Functional Gastrointestinal Disorders [10]. The questionnaire included demographic information, main intestinal symptoms, defecation-related symptoms, extraintestinal symptoms, psychological status, sleeping status and diet. The questionnaires were administered by specially trained researchers via face-to-face interviews and were re-checked by other specialists. The main intestinal symptoms and defecation-related symptoms were scored according to frequency and degree (Table 1). The overall score of intestinal symptoms was calculated based on a possible score of 36. Because pre-defecation abdominal pain/discomfort is the core symptom of IBS, it was scored separately according to degree and frequency.

**Table 1:** **Scoring of intestinal symptoms**

**Scores (point)**	**0**	**1**	**2**	**3**
**Intestinal symptoms**				
***Main intestinal symptoms***				
Frequency of pre-defecation abdominal pain/discomfort	–	≥3 day/month	≥1 day/week	Every day
Degree of pre-defecation abdominal pain/discomfort	–	Mild	Moderate	Severe
Frequency of bowel movement during symptom onset	–	≤3/day	4-5/day	≥6/day
Stool form during symptom onset (Bristol stool form scale)	–	4-5	6	7
Improvement of abdominal pain/discomfort with defecation	–	Complete relief	Relief ≥1/2	Relief <1/2
***Defecation-related symptoms***				
Degree of abdominal distention	None	Mild	Moderate	Severe
Degree of urgency	None	Mild	Moderate	Severe
Degree of defecation straining	None	Mild	Moderate	Severe
Frequency of passing mucus	None	Occasionally	Sometimes	Often
Degree of incomplete defecation	None	Mild	Moderate	Severe
Amount of fecal incontinence	None	Small	Medium	Large

#### Simplified Chinese version of the Irritable Bowel Syndrome-Quality of Life (IBS-QOL) questionnaire

The simplified Chinese version [11] of the IBS-QOL questionnaire [12] was utilized. This scale has been validated and proven suitable for evaluating QOL in Chinese IBS patients. This scale includes 34 items, each with a five-point Likert-type response scale ranging from 1 (not at all) to 5 (a great deal). The individual responses to the 34 items were summed and averaged for a total score and then transformed to a 0-100 scale for ease of interpretation, with higher scores indicating better IBS-specific QOL. There are also eight domain scores, with the total score of each also summed and transformed to a 0-100 scale [13]. The mean overall score in healthy Chinese subjects is 95.50 ± 6.73, with the scores on each of the eight domains being ≥ 90.00 [11].

#### Hamilton Depression Scale and Hamilton Anxiety Scale

Psychological status was evaluated using the Hamilton Depression Scale (HAMD) and the Hamilton Anxiety Scale (HAMA) [14], which were administered by two specially trained professionals through conversation and observation. The HAMD scale was divided into five structural factors: anxiety/somatization, cognitive disorder, psychomotor retardation, sleep disorder and weight; and the HAMA scale was divided into two structural factors: psychic anxiety and somatic anxiety.

### Statistical analysis

Patient data were entered into a database established using Epidata software version 3.02. Data were analyzed using SPSS 18.0 software (SPSS, Chicago, IL, USA). Continuous data are reported as mean ± standard deviation and categorical data as number (rate). Continuous variables were analyzed using ANOVA and the independent two-sample *t*-test or Mann-Whitney test. Categorical variables were compared using the chi-squared test and the Mantel-Haenszel test. Correlation analysis was performed using Spearman rank correlation analysis or multiple linear regression analysis. A p value <0.05 was considered statistically significant.

## Results

### General data

This study included 227 IBS-D patients, 133 males and 94 females (male/female ratio 1.41:1), of mean age 44.68 ± 10.81 years and the mean duration of disease 7.00 ± 7.32 years.

### QOL in IBS-D patients

The overall IBS-QOL score was 71.68 ± 18.54. The lowest score was for food avoidance (53.71 ± 26.92), the second lowest was for dysphoria (64.98 ± 24.40) and the highest was for body image (86.18 ± 15.67). Females had significantly lower food avoidance (49.38 ± 26.50 vs. 56.77 ± 26.89, p = 0.037) and social reaction (76.53 ± 22.65 vs. 83.41 ± 17.94, p = 0.015) scores than males (Table 2). Significant differences between genders were observed on six items of the IBS-QOL questionnaire (Table 3). In contrast, patient age, occupation, education, labor type (manual or mental), marriage and family economic status did not significantly affect their QOL.

**Table 2:** **IBS-QOL and eight domain scores in male and female IBS-D patients**

**Score**	**Total (N = 227)**	**Male (N = 133)**	**Female (N = 94)**	p value*
***IBS-QOL***	71.68 ± 18.54	73.76 ± 17.29	68.73 ± 19.89	0.076
Dysphoria	64.98 ± 24.40	66.71 ± 23.23	62.53 ± 25.90	0.275
Interference with activity	67.94 ± 23.79	70.62 ± 21.68	64.13 ± 26.13	0.105
Body image	86.18 ± 15.67	88.25 ± 13.61	83.24 ± 17.85	0.076
Health worry	67.40 ± 22.71	68.48 ± 22.34	65.87 ± 23.25	0.427
Food avoidance	53.71 ± 26.92	56.77 ± 26.89	49.38 ± 26.50	0.037†
Social reaction	80.56 ± 20.27	83.41 ± 17.94	76.53 ± 22.65	0.015†
Sexual	83.09 ± 25.22	83.65 ± 24.96	82.31 ± 25.69	0.706
Relationship	81.75 ± 20.46	83.39 ± 19.07	79.43 ± 22.19	0.232

*Males vs. females; †p < 0.05.

**Table 3:** **Significant between gender differences on six items of the IBS-QOL questionnaire**

**Item**	**Male (N = 133)**	**Female (N = 94)**	p value*
**Q2** I am embarrassed by the smell caused by my bowel problems	81.39 ± 21.81	72.87 ± 30.16	0.029
**Q17** I worry that people think I exaggerate my bowel problems	84.21 ± 26.29	75.00 ± 31.75	0.018
**Q21** My bowel problems limit what I call wear	92.67 ± 19.65	78.72 ± 32.78	0.000
**Q22** I have to avoid strenuous activity because of my bowel problems	81.95 ± 25.44	73.40 ± 32.13	0.026
**Q27** Long trips are difficult for me because of my bowel problems	68.61 ± 33.28	55.05 ± 37.13	0.004
**Q28** I feel frustrated that I cannot eat when I want because of my bowel problems	65.98 ± 31.73	57.18 ± 34.71	0.049

*Males vs. females.

### The impact of intestinal symptom scores on IBS-QOL

The overall intestinal symptoms score was 15.25 ± 3.30. There were no significant between gender differences in overall intestinal symptoms scores, main intestinal symptom scores and defecation-related symptom scores (all p > 0.05). Overall IBS-QOL scores correlated negatively with the overall intestinal symptom scores (*r* = -0.342, p = 0.000), main intestinal symptom scores (*r* = -0.218, p = 0.001) and defecation-related symptom score (*r* = -0.314, p = 0.000).

### The impact of HAMD and HAMA scores on IBS-QOL

**Figure 1:**
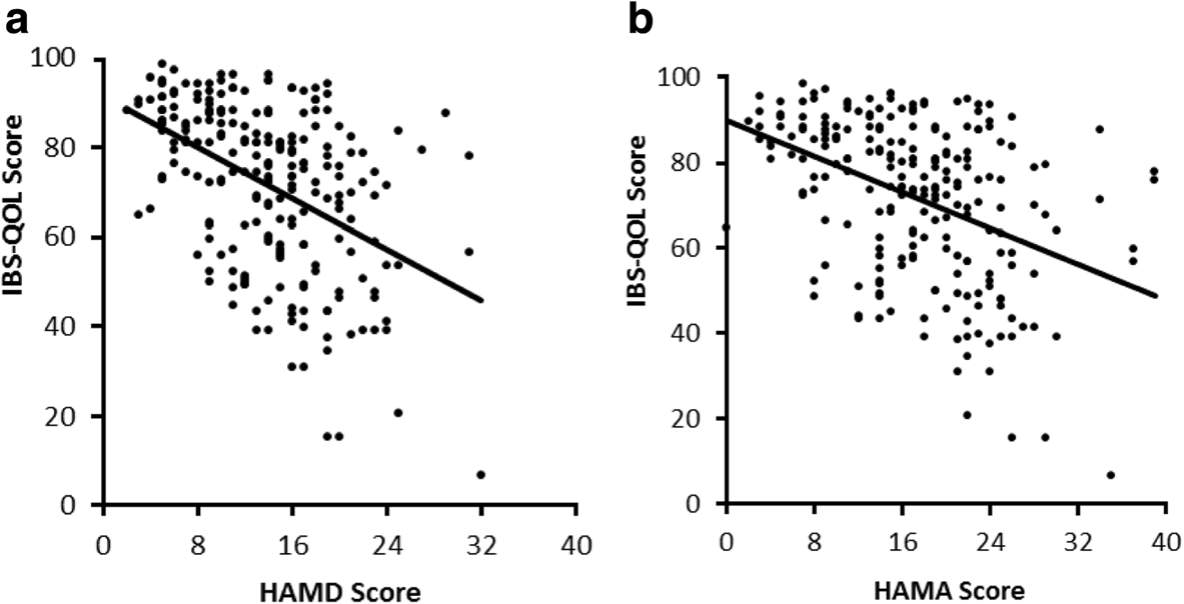
Correlation between overall IBS-QOL scores and scores on the **(a)** HAMD and **(b)** HAMA. Overall IBS- QOL scores were significantly negatively correlated with HAMD (r_1_ = -0.460, p = 0.000) and HAMA (r_2_ = -0.434, p = 0.000) scores.

The mean HAMD score was 13.92 ± 5.90 and the mean HAMA score was 17.19 ± 7.37. Both HAMD score [*r*_1_ = -0.460, p = 0.000 (Figure 1a)] and HAMA score [r_2_ = -0.434, p = 0.000 (Figure 1b)] showed significant negative correlations with overall IBS-QOL score.

Comparison of the structural factors in the scales and overall scores of HAMD and HAMA between male and female patients showed that female patients had higher scores than male patients on both the HAMD and HAMA; as well as higher scores on the structural factors of the HAMD scale, including anxiety/somatization, cognitive disorder, and psychomotor retardation; and higher scores on the structural factors of the HAMA scale, including somatic anxiety and psychic anxiety (p < 0.01 each; Table 4).

**Table 4:** **Comparison of HAMD, HAMA and structural factors scores between male and female IBS-D patients**

**Score**	**Total (N = 227)**	**Male (N = 133)**	**Female (N = 94)**	p value*
***HAMD***	13.92 ± 5.90	12.75 ± 5.24	15.56 ± 6.40	0.000†
Anxiety /somatization	7.49 ± 2.75	6.98 ± 2.56	8.23 ± 2.85	0.001†
Cognitive disorder	1.57 ± 1.34	1.35 ± 1.61	1.86 ± 1.49	0.005†
Psychomotor retardation	2.61 ± 1.87	2.35 ± 1.61	2.98 ± 2.13	0.012†
Sleep disorder	2.14 ± 1.80	1.96 ± 1.68	2.38 ± 1.95	0.083
Body weight	0.11 ± 0.34	0.11 ± 0.33	0.11 ± 0.34	0.980
***HAMA***	17.19 ± 7.37	15.53 ± 6.38	19.54 ± 8.05	0.000†
Somatic anxiety	8.64 ± 4.14	7.82 ± 3.62	9.79 ± 4.54	0.000†
Psychic anxiety	8.55 ± 3.95	7.71 ± 3.61	9.74 ± 4.12	0.000†

*Males vs. females; †p < 0.05.

### Relationship between intestinal symptom scores and HAMD and HAMA scores

Linear correlations were observed between the overall score of intestinal symptoms and the HAMD (r_1_ = 0.316, p = 0.000) and HAMA (r_2_ = 0.303, p = 0.000) scores. In addition, significant linear correlations were observed between main intestinal symptom scores and HAMD (r_1_ = 0.214, p = 0.000) and HAMA (r_2_ = 0.191, p = 0.004) scores, as well as between defecation-related symptom scores and HAMD (*r*_1_ = 0.285, p = 0.000) and HAMA (r_2_ = 0.280, p = 0.000) scores.

### The impact of intestinal symptoms, depression and anxiety on the IBS-QOL

The collinearity of 18 items, including 11 intestinal symptom scores, five structural factor scores of the HAMD scale, and two structural factor scores of the HAMA scale was analyzed. Five independent factors were identified, including the frequency of pre-defecation abdominal pain/discomfort, degree of urgency, frequency of passing mucus, psychomotor retardation and somatic anxiety. Multiple linear regression analysis showed that the degree of defecation urgency, frequency of passing mucus and psychomotor retardation were independent predictors of decreased IBS-QOL (Table 5). When independent predictors were calculated separately for male and female patients, we found that the degree of urgency, frequency of passing mucus, somatic anxiety and psychomotor retardation were independent predictors of a low overall IBS-QOL score for male patients; whereas psychomotor retardation was the only independent predictor of low overall IBS-QOL score for female patients (Table 6).

**Table 5:** **Factors independently predictive of poorer quality of life in IBS-D patients**

**Items**	**Regression**	**p value**	**Partial correlation**		
	**coefficient (β)**		**Coefficient**	**p value**	
Constants	91.573	0.000			
Degree of xurgency	-2.725	0.006	-0.157	0.019	R^2^ = 0.270
Frequency of passing mucus	-2.412	0.015	-0.146	0.029	
Psychomotor retardation	-4.327	0.000	-0.298	0.000

**Table 6:** **Factors independently predictive of reduced quality of life in male and female IBS-D patients**

**Items**	**Regression coefficient (β)**	**p value**	**Partial correlation**		
			**Coefficient**	**p value**	
***Male***					
Constants	96.651	0.000			
Degree of urgency	-2.733	0.022	-0.181	0.041	R^2^ = 0.264
Frequency of passing mucus	-2.947	0.014	-0.207	0.019	
Psychomotor retardation	-2.860	0.005	-0.226	0.010	
Somatic anxiety	-0.927	0.039	-0.199	0.024	
***Female***					
Constants	82.910	0.000			
Psychomotor retardation	-4.759	0.000	-0.376	0.000	R^2^ = 0.252

## Discussion

Being specific for IBS, the IBS-QOL scale has been widely used in clinical practice [4,15-17] and has been shown suitable for evaluating the severity of IBS and assessing treatment outcomes in clinical practice [17]. The present study found that overall IBS-QOL scores were generally lower in IBS-D patients, with food avoidance scores being the lowest. This result is in accordance with the results of other studies evaluating IBS patients [3,4,8,13,17-19]. Epidemiological studies in both Asian [7,20-22] and Western [23-26] patients have shown that the prevalence of IBS was higher in females than in males. In addition, female patients were reported to have lower IBS-QOL scores than males [15,27]. In the present study, 58.6% of the patients were males, suggesting that males may be more likely to have IBS-D whereas females are more likely to have IBS-C [28,29], or that the rate of hospital visits may be higher among Chinese males than females with IBS-D. Although overall IBS-QOL scores were similar in males and females, food avoidance and social reaction scores were lower in females, similar to previous findings [3,16]. The significantly lower food avoidance scores in female IBS-D patients were due mainly to their response to the statement, “I have to watch the amount of food I eat because of my bowel problems.” This suggests that female IBS-D patients are more likely to regard a certain type of improper food as the main cause of their intestinal symptoms and want to relieve their symptoms by avoiding that type of food. In contrast, male IBS patients are reported to have different IBS-related types and quantities of food than females [30]. The social reaction scores were significantly lower for female than male IBS-D patients, as indicated by their replies to the statements: “I am embarrassed by the smell caused by my bowel problems” and “I worry that people think I exaggerate my bowel problems”, to which female patients responded more affirmatively. In addition, female IBS-D patients were more likely to regard their bowel problems as limiting the clothing they could wear (item 21) and to want to avoid strenuous activity (item 22) and long trips (item 27). All of these responses indicate that the social reactions of female IBS-D patients are affected by their intestinal symptoms. Although gender-related traits have been associated with IBS-QOL, it is unclear whether these traits or their linked psychosocial disorders affected patients’ responses to their intestinal symptoms [15].

Studies of the impact of age, type of work, occupation, education, marriage and family economic status on the QOL of IBS patients have yielded different results [7,21,31-33]. A study in Egypt showed that people with low education and income levels and those performing manual labor were more likely to suffer from IBS than educated professionals [34]. In contrast, this study found that QOL in Chinese patients with IBS-D was not affected by age, education, occupation, type of work, marriage or family economic status, consistent with findings in Iran [4].

Korean studies [3,5] showed that the overall score of intestinal symptoms and the severity described by the patient were independent factors affecting health-related QOL (HRQOL) scores of IBS patients, and that pre-defecation abdominal pain/discomfort significantly affected patients’ QOL [3,35,36]. In the present study, overall IBS-QOL scores of IBS-D patients were significantly correlated with overall scores of intestinal symptoms. Further analysis showed that the main intestinal symptoms, including the frequency and degree of pre-defecation abdominal pain/discomfort, increased numbers of bowel movements and loose/watery stools at symptom onset, and improvements in abdominal pain/discomfort following defecation, did not significantly affect the QOL of patients with IBS-D. Although no single symptom significantly affected the QOL of IBS-D patients, a significant negative correlation was observed between their main intestinal symptom scores and overall IBS-QOL scores, indicating that the integrated severity of intestinal symptom may reduce patients’ QOL.

Defecation urgency and passing mucus were the most common IBS-associated symptoms in the present study, with both being independent risk factors affecting QOL. We did not determine the mechanism by which urgency and passing mucus affected patients’ QOL. In clinical practice, patients usually state that urgency limited their travel activities, even for taking a bus to working sites. Most people in China use public transportation, with few sites on these routes having toilets. The effect of passing mucus on QOL may be related to patients’ lack of medical knowledge and paying too much attention to passing mucus. Urgency and passing mucus contributed to the decreased overall IBS-QOL score in male patients and had a greater effect on total QOL scores of male than of female IBS-D patients.

Psychological factors are also associated with the pathogenesis of IBS, since they can affect intestinal function via the autonomic nervous system and brain-gut axis [37,38]. It has been estimated that 50% to 90% of IBS patients have psychological disorders [38,39], primarily depression and anxiety, although neuroses and hypochondria have also been reported. These comorbid psychological disorders may affect patients’ QOL [3,6,13,40], and psychological treatment may improve their QOL [11,41]. Evaluation using the Hospital Anxiety and Depression Scale (HAD) showed that IBS patients with anxiety and depression often had more serious intestinal symptoms and that their QOL was compromised significantly [5,42]. However, few studies have analyzed the structural factors linking the HAD and HAMA/HAMD scales with patients’ QOL. One study found that eight factors independently predicted mental HRQOL, including feeling tense, nervous, and hopeless; difficulty sleeping; tiring easily; low sexual interest; IBS symptom interference with sexual function; and low energy [40]. Results of the present study showed that overall IBS-QOL scores correlated negatively with HAMD and HAMA scores, and that psychomotor retardation on the HAMD scale was able to predict patients’ QOL independently. This factor on the HAMD scale includes depressed mood (dysphoria), work and interests, psychomotor retardation status and sexual function (loss of libido). Depressed mood and loss of interest are core symptoms in depressive disorders. Depressed patients with psychomotor retardation may require intensive care to ensure adequate food and fluid intake and sufficient personal care. Our results indicate the need to recognize psychomotor retardation and to apply appropriate intervention to improve QOL.

HAMD scores, including scores on its structural factors anxiety/somatization, cognitive disorder, and psychomotor retardation, and HAMA scores, including scores of its two structural factors, somatic anxiety and psychic anxiety, were significantly higher in female than in male patients with IBS-D, suggest that the associated psychological disorders were more significant in female patients. Only the psychomotor retardation score, an acknowledged characteristic of depression, significantly affected patients’ QOL. However, somatic anxiety was an independent predictor of QOL in male patients.

The associations between intestinal symptoms and/or the psychological state of IBS patients and their QOL have been verified [5,6,36,43]. Psychological distress is less dependent on gastrointestinal symptom severity in patients with IBS than in those with inflammatory bowel diseases; in the latter, psychological distress has a greater direct effect on HRQOL than gastrointestinal symptoms [6]. Results of the present study showed that the intestinal symptom scores of IBS-D patients were positively correlated with HAMD/HAMA scores, but that both were negatively correlated with patients’ IBS-QOL scores, suggesting an interactive network linking intestinal symptoms, QOL, and psychological state. These findings suggest that the frequent onset or continuous presence of intestinal symptoms (represented by high intestinal symptom score) can aggravate patients’ psychological burden, and that comorbid psychological disorders may aggravate patients’ subjective feelings about their intestinal and somatic symptoms, resulting in multiple extraintestinal symptoms. This may lead patients to excessively avoid foods and disproportionately worry about their intestinal symptoms, reducing their outside activities and, in turn, their QOL. This cycle suggests that the reduced QOL in IBS-D patients results from the combined impact of intestinal symptoms and psychological abnormalities.

This study had several limitations. First, since our hospital is a referral center for patients in China with difficult and complicated diseases, most of the patients enrolled in this study had moderate to severe illness. Second, the IBS symptom questionnaire and the intestinal symptom scoring method have not been validated. Finally, the severity and frequency of intestinal and defecation-related symptoms were reported by patients themselves. However, nociceptive contributions, the degree of disability, culture, psychological distress and poor HRQOL may influence patients’ report of the severity of their symptoms [35].

## Conclusions

The combination of intestinal symptoms and comorbid psychological factors in patients with IBS-D affects their QOL, with the impact of both differing by gender. In female patients, comorbid psychological disorders are more significant, and the effects of food avoidance and social reactions are more obvious. Therefore, it is recommended that intestinal and defecation-related symptoms be controlled promptly in male patients and that psychological interventions be applied to female patients with obvious anxiety and/or depression.

## Abbreviations

IBS: Irritable bowel syndrome
IBS-D: Irritable bowel syndrome with diarrhea
QOL: Quality of life
IBS-QOL: Irritable bowel syndrome-quality of life
HAMD: Hamilton depression scale
HAMA: Hamilton anxiety scale
HRQOL: Health-related quality of life
HAD: Hospital anxiety and depression scale
